# Prognostic Prediction Value of qSOFA, SOFA, and Admission Lactate in Septic Patients with Community-Acquired Pneumonia in Emergency Department

**DOI:** 10.1155/2020/7979353

**Published:** 2020-04-06

**Authors:** Haijiang Zhou, Tianfei Lan, Shubin Guo

**Affiliations:** ^1^Department of Emergency Medicine, Beijing Chao-yang Hospital, Capital Medical University, Beijing Key Laboratory of Cardiopulmonary Cerebral Resuscitation, Beijing, China; ^2^Department of Allergy, Beijing Shijitan Hospital, Capital Medical University, Beijing, China

## Abstract

**Background:**

Community-acquired pneumonia (CAP) is a leading cause of sepsis and common presentation to emergency department (ED) with a high mortality rate. The prognostic prediction value of sequential organ failure assessment (SOFA) and quick SOFA (qSOFA) scores in CAP in ED has not been validated in detail. The aim of this research is to investigate the prognostic prediction value of SOFA, qSOFA, and admission lactate compared with that of other commonly used severity scores (CURB65, CRB65, and PSI) in septic patients with CAP in ED.

**Methods:**

Adult septic patients with CAP admitted between Jan. 2017 and Jan. 2019 with increased admission SOFA ≥ 2 from baseline were enrolled. The primary outcome was 28-day mortality. The secondary outcome included intensive care unit (ICU) admission, mechanical ventilation, and vasopressor use. Prognostic prediction performance of the parameters above was compared using receiver operating characteristic (ROC) curves. Kaplan–Meier survival curves were compared using optimal cutoff values of qSOFA and admission lactate.

**Results:**

Among the 336 enrolled septic patients with CAP, 89 patients died and 247 patients survived after 28-day follow-up. The CURB65, CRB65, PSI, SOFA, qSOFA, and admission lactate levels were statistically significantly higher in the death group (*P* < 0.001). qSOFA and SOFA were superior and the combination of qSOFA + lactate and SOFA + lactate outperformed other combinations of severity score and admission lactate in predicting both primary and secondary outcomes. Patients with admission qSOFA < 2 or lactate ≤ 2 mmol/L showed significantly prolonged survival than those patients with qSOFA ≥ 2 or lactate > 2 mmol/L (log-rank *χ*^2^ = 59.825, *P* < 0.001). The prognostic prediction performance of the combination of qSOFA and admission lactate was comparable to the full version of SOFA (AUROC 0.833 vs. 0.795, *Z* = 1.378, *P*=0.168 in predicting 28-day mortality; AUROC 0.868 vs. 0.895, *Z* = 1.022, *P*=0.307 in predicting ICU admission; AUROC 0.868 vs. 0.845, *Z* = 0.921, *P*=0.357 in predicting mechanical ventilation; AUROC 0.875 vs. 0.821, *Z* = 2.12, *P*=0.034 in predicting vasopressor use).

**Conclusion:**

qSOFA and SOFA were superior to CURB65, CRB65, and PSI in predicting 28-day mortality, ICU admission, mechanical ventilation, and vasopressor use for septic patients with CAP in ED. Admission qSOFA with lactate is a convenient and useful predictor. Admission qSOFA ≥ 2 or lactate > 2 mmol/L would be very helpful in discriminating high-risk patients with a higher mortality rate.

## 1. Introduction

Community-acquired pneumonia (CAP), a major cause of sepsis and the 8th leading cause of death, is a common respiratory tract infection encountered in the emergency department (ED) [1]. CAP is caused by virus, bacteria, or fungi, and its symptoms include cough, chest pain, fever, and dyspnea. Diagnosis of CAP is determined by symptoms, physical examinations, laboratory findings, and chest radiographs, as well as etiologic agent culture. Severity evaluation is of vital importance for treatment location selection, empirical antimicrobial initiation, and adjunctive and supportive treatment adoption [2]. For CAP severity assessment, the CURB65 (confusion, urea > 7 mmol/L, respiratory rate ≥ 30/min, blood pressure < 90 mmHg systolic and/or ≤60 mmHg diastolic, and age ≥ 65 years) and pneumonia severity index (PSI) score are most widely used worldwide [3, 4]. Preliminary research studies demonstrated that there were no significant differences in overall test performance between CURB65 and PSI [5].

CAP accounts for substantial mortality worldwide, with a high risk of developing respiratory failure and septic shock. The third international consensus definitions for sepsis and septic shock generated new definitions and updated the clinical criteria [6]. Sepsis was defined as life-threatening organ dysfunction caused by a dysregulated host response to infection, and organ dysfunction was defined as an increase in the sequential organ failure assessment (SOFA) score of 2 or higher [6], highlighting the clinical implication of (SOFA) score and qSOFA (respiratory rate ≥ 22/min, altered mentation, and systolic blood pressure ≥ 100 mmHg) score [6]. Lactate has been utilized as a marker to evaluate the acid-base homeostasis and perfusion status of patients. Higher lactate levels are reported to be associated with higher hospital mortalities and longer length of ED and hospital stay in unselected patients [7, 8]. However, the prognostic value of SOFA score, qSOFA score, and lactate in patients with CAP in the emergency department (ED) has not been fully elucidated.

In the present study, we explored the prognostic prediction value of SOFA, qSOFA, and admission lactate in patients with CAP in ED, and the results were compared with that of other commonly used CAP severity scores (CURB65, CRB65, PSI).

## 2. Patients and Methods

This was a single-center, retrospective cohort study carried out in Beijing Chao-yang Hospital, Capital Medical University, which is a tertiary referral hospital located in the northern region of China with approximately 250,000 annual ED visits per year. The Institutional Review Board and Medical Ethics Committee have approved this study, and written informed consent was waived because of the retrospective design of this study.

Adult patients with the discharge diagnosis of CAP admitted between Jan. 2017 and Jan. 2019 were screened. The inclusion criteria were age ≥ 18 years, new infiltrates on chest radiograph, and two or more symptoms including cough, fever, dyspnea, sputum production, breathlessness, and/or chest pain [9]. In accordance with sepsis 3.0 criteria, we only enrolled CAP patients with increased SOFA score ≥ 2 from baseline. The following patients were excluded from this study: (1) age < 18 years; (2) patients with metastatic tumor, acquired immunodeficiency syndrome (AIDS), active tuberculosis, previous transplantation, immunosuppressive therapy, and pregnancy; (3) patients transferred from other hospitals or diagnosed with hospital acquired pneumonia; (4) patients with incomplete clinical, laboratory, or radiographic records; (5) patients with increased admission SOFA score < 2 from baseline.

Demographic characteristics of all enrolled patients on ED arrival were collected and recorded by trained triage nurses on admission. Past history, comorbidities, and vital signs (blood pressure, heart rate, breath rate, and state of consciousness) were also recorded. Laboratory parameters on admission including full blood count, hemoglobin level (HGB), hemocrit (HCT), platelet level (PLT), albumin (ALB), hepatic function (aspartate aminotransferase (AST), alanine aminotransferase (ALT), total bilirubin (TBIL), direct bilirubin (DBIL)), renal function (creatinine (CREA), blood urea nitrogen (BUN)), electrolytes, and arterial blood gas including lactate level were assessed and collected. CURB65, CRB65, PSI, SOFA, and qSOFA scores on admission for each patient were calculated according to international criteria and analyzed, utilizing data collected on ED arrival.

All patients were followed up for 28 days through medical records, and 28-day mortality was the primary study end point. According to their prognosis after 28-day admission, patients were divided into death group and survival group. The secondary outcome included ICU admission, mechanical ventilation use, and vasopressor use.

All analyses were performed using SPSS 22.0 statistical software package (SPSS Inc, Chicago, IL, USA). Data with normal distribution were described as mean ± standard deviation and compared using Student's *t*-test. Data with skewed distribution were expressed as median (interquartile range) and compared using the Mann–Whitney *U* nonparametric test. The categorical variables were described as percentages and compared using the chi-squared test or Fisher's exact test. Receiver operating characteristics (ROC) curves for each predictor were constructed, and the area under the curve (AUC) was determined to assess their predictive values. Combination models of severity score and lactate were established using several logistic regressions to save the predicted probabilities. ROC curve analysis was performed using the saved probabilities as a new indicator. Comparisons of each predictor were conducted using MedCalc 15.0 Software (Acacialaan, Ostend, Belgium). A *Z*-test was used for comparing the AUCs between different curves. For comparison of the AUCs, *Z* = (A_1_−A_2_)/$\sqrt{{\left({\text{SE}}_{1}\right)}^{2}+{\left({\text{SE}}_{2}\right)}^{2}}$ was used, the test values being *Z*_0.05_ = 1.96 and *Z*_0.01_ = 2.58. Based on the cutoff values, sensitivity, specificity, positive predictive value (PPV), and negative predictive value (NPV) were also calculated. Kaplan–Meier survival curves were drawn using cutoff values of qSOFA and lactate. A two-tailed value of *P* < 0.05 was considered statistically significant.

## 3. Results

**Figure 1:**
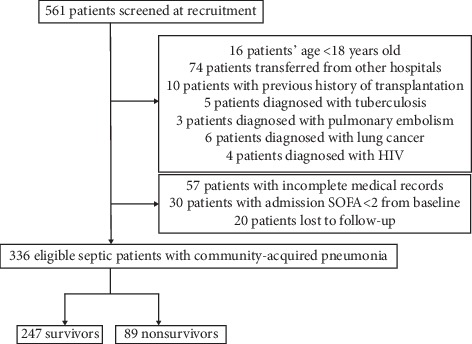
Flow chart of patients enrolled in our study.

A total of 561 patients were screened at recruitment, and 225 patients were excluded (Figure 1). A total of 336 patients were finally included in our study group. Of the 225 excluded patients, 16 patients' age < 18 years old, 74 patients were transferred from other hospitals, 10 patients were with a history of previous transplantation, 5 patients were finally diagnosed with pulmonary tuberculosis, 3 patients were with pulmonary thromboembolism, 6 patients were with lung cancer, 4 patients were with HIV, 57 patients were with incomplete medical records, 30 patients were with admission SOFA score < 2 from baseline, and 20 patients were lost to follow-up with unknown prognosis.

Of the 336 patients, 89 patients were dead and 247 patients survived after 28-day follow-up, and the total mortality rate was 26.5% (Table 1). The total mean age was 76 (61, 84) years, and the male-to-female ratio was 1.73 : 1. Comorbidities of enrolled patients include chronic obstructive pulmonary disease (COPD) (11.9%), cardiovascular disease (CDVD) (14.3%), cerebrovascular disease (CBVD) (26.2%), diabetes (22.9%), chronic renal disease (CRD) (8.9%), and hepatobiliary disease (HBD) (7.1%). There were no significant differences between the death and survival groups in age (*P*=0.136) and male-female ratio (*P*=0.914). CBVD was the most common comorbidity, and a previous history of diabetes mellitus was more common among nonsurvivors (Table 1). 61 patients were admitted in ICU, and their age was older than those who were not admitted in ICU (Table 1). As to laboratory parameters, the ALB level was significantly lower, while the creatine and blood urea nitrogen (BUN) levels were significantly higher in the death group, the patients admitted in ICU, and the patients who use mechanical ventilation or vasopressors, respectively (*P* < 0.05) (Tables 1 and 2). The vital signs of the nonsurvivors, the patients admitted in ICU, and the patients used mechanical ventilation or vasopressors were more unstable (*P* < 0.05) (Tables 1 and 2). The CURB65, CRB65, PSI, SOFA, qSOFA, and admission lactate levels were significantly higher in the death group, the ICU admission group, the mechanical ventilation group, and the vasopressors use group (*P* < 0.05) (Tables 1 and 2).

**Figure 2:**
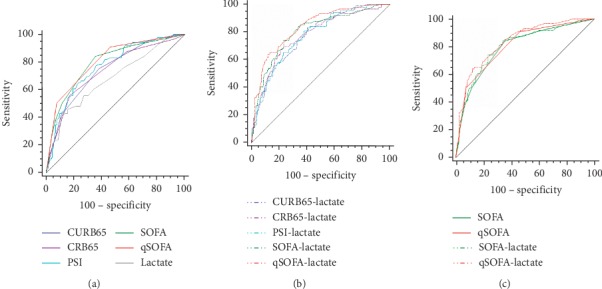
ROC curve comparisons of (a) CURB65, CRB65, PSI, SOFA, qSOFA, and admission lactate in predicting 28-day mortality; (b) different combinations of CAP severity scores and admission lactate in predicting 28-day mortality; and (c) SOFA, qSOFA, SOFA + lactate, and qSOFA + lactate in predicting 28-day mortality.

In predicting 28-day mortality, ROC curve comparisons showed that the AUROC of qSOFA (0.807) was the highest among single predictors (Table 3, Figure 2), followed by SOFA (0.795), PSI (0.768), CURB65 (0.744), CRB65 (0.737), and admission lactate (0.679). Moreover, the qSOFA score was with the highest sensitivity (91%) and negative predictive value (NPV) (94.3%), which highlighted the prediction performance of qSOFA. However, among the combinations of severity score and admission lactate, the AUROC of qSOFA + lactate (0.833) was the highest, followed by the combination of SOFA + lactate (0.803), CRB65 + lactate (0.776), PSI + lactate (0.774), and CURB65 + lactate (0.772). Multiple pairwise comparisons among single predictors showed that there were no significant differences between qSOFA and SOFA (*Z* = 0.373, *P*=0.709), qSOFA and PSI (*Z* = 1.296, *P*=0.195), CURB65 and PSI (*Z* = 0.940, *P*=0.347), CURB65 and SOFA (*Z* = 1.499, *P*=0.134), and PSI and SOFA (*Z* = 0.835, *P*=0.404). There were significant differences between qSOFA and CURB65 (*Z* = 2.333, *P*=0.020), qSOFA and CRB65 (*Z* = 2.504, *P*=0.012), qSOFA and lactate (*Z* = 3.246, *P*=0.001), and SOFA and lactate (*Z* = 3.143, *P*=0.002), whereas pairwise comparisons among combinations of severity scores and lactate demonstrated that the combination of qSOFA + lactate demonstrated superiority over other combinations except the combination of SOFA + lactate. There were no significant differences between CURB65 + lactate and CRB65 + lactate (*Z* = 0.244, *P*=0.807), CURB65 + lactate and PSI + lactate (*Z* = 0.062, *P*=0.951), CURB65 + lactate and SOFA + lactate (*Z* = 1.054, *P*=0.292), PSI + lactate and SOFA + lactate (*Z* = 0.993, *P*=0.321), and SOFA + lactate and qSOFA + lactate (*Z* = 1.187, *P*=0.235).

**Figure 3:**
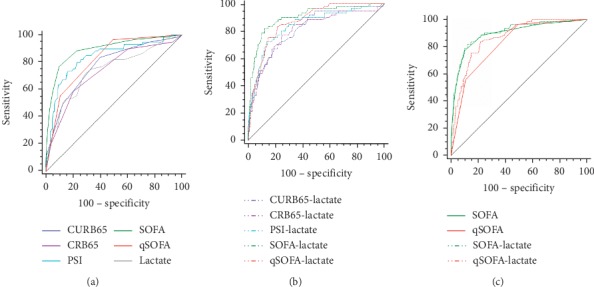
ROC curve comparisons of (a) CURB65, CRB65, PSI, SOFA, qSOFA, and admission lactate in predicting ICU admission; (b) different combinations of CAP severity scores and admission lactate in predicting ICU admission; and (c) SOFA, qSOFA, SOFA + lactate, and qSOFA + lactate in predicting ICU admission.

The ability to predict ICU admission was higher when the SOFA was used rather than either CURB65, CRB65, qSOFA, or PSI (Table 4, Figure 3). These differences were statistically significant. SOFA achieved the highest AUROC (0.895) in predicting ICU admission, followed by PSI (0.837), qSOFA (0.822), CURB65 (0.774), lactate (0.742), and CRB65 (0.738). As to the comparisons of combinations of severity scores and lactate, SOFA + lactate achieved the highest AUROC (0.902), followed by qSOFA + lactate (0.868), PSI + lactate (0.840), CURB65 + lactate (0.818), and CRB65 + lactate (0.806). There were no significant differences between SOFA + lactate and qSOFA + lactate (*Z* = 1.476, *P*=0.140) and SOFA + lactate and PSI + lactate (*Z* = 1.949, *P*=0.051).

**Figure 4:**
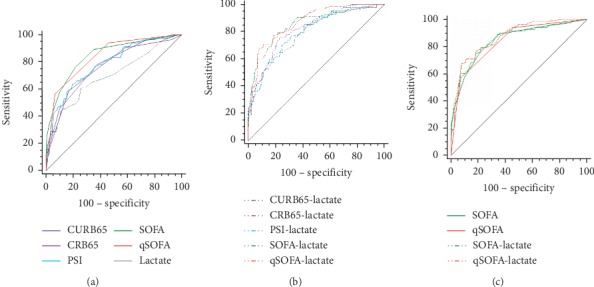
ROC curve comparisons of(a) CURB65, CRB65, PSI, SOFA, qSOFA, and admission lactate in predicting mechanical ventilation; (b) different combinations of CAP severity scores and admission lactate in predicting mechanical ventilation; and (c) SOFA, qSOFA, SOFA + lactate and qSOFA + lactate in predicting mechanical ventilation.

**Figure 5:**
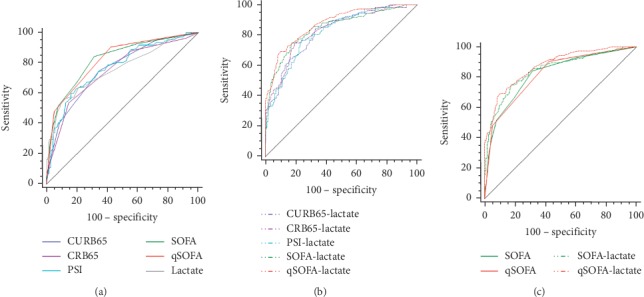
ROC curve comparisons of(a) CURB65, CRB65, PSI, SOFA, qSOFA, and admission lactate in predicting vasopressor use; (b) different combinations of CAP severity scores and admission lactate in predicting vasopressor use; and (c) SOFA, qSOFA, SOFA + lactate, and qSOFA + lactate in predicting vasopressor use.

In predicting mechanical ventilation (Table 5, Figure 4), SOFA achieved the highest AUROC (0.845), followed by qSOFA (0.838), PSI (0.780), CURB65 (0.771), and CRB65 (0.758). SOFA and qSOFA had similar prediction performance (*Z* = 0.215, *P*=0.830). As to the combinations of severity scores and lactate, SOFA + lactate (AUROC = 0.851) and qSOFA + lactate (AUROC = 0.868) also had similar prediction value (*Z* = 0.746, *P*=0.456) and demonstrated superiority over other combinations. Among the single predictors in predicting vasopressor use (Table 6, Figure 5), the AUROC of SOFA (0.821) and qSOFA (0.820) was higher than that of PSI (0.767), CURB65 (0.759), CRB65 (0.746), and lactate (0.758), while the difference between SOFA and qSOFA was not statistically significant (*Z* = 0.046, *P*=0.964). In terms of the combinations of severity scores and lactate, SOFA + lactate (AUROC = 0.848) and qSOFA + lactate (AUROC = 0.875) also had similar prediction value (*Z* = 1.426, *P*=0.154) and demonstrated superiority over other combinations.

As to the comparison of the prediction ability of SOFA and the combination of qSOFA and admission lactate, no significant differences were found between them in predicting 28-day mortality (0.795 vs. 0.833, *Z* = 1.378, *P*=0.168), ICU admission (0.895 vs. 0.868, *Z* = 1.022, *P*=0.307), and mechanical ventilation (0.845 vs. 0.868, *Z* = 0.921, *P*=0.357), while qSOFA + lactate was significantly superior to SOFA in predicting vasopressor use (0.821 vs. 0.875, *Z* = 2.12, *P*=0.034).

**Figure 6:**
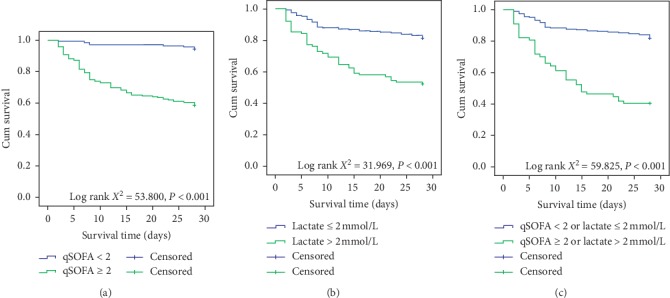
Kaplan–Meier survival curve comparison: (a) between septic CAP patients with admission qSOFA < 2 and septic CAP patients with admission qSOFA ≥ 2; (b) between septic CAP patients with admission lactate ≤ 2 mmol/L and septic CAP patients with admission lactate > 2 mmol/L; (c) between septic CAP patients with admission qSOFA < 2 or lactate ≤ 2 mmol/L and septic CAP patients with admission qSOFA ≥ 2 or lactate > 2 mmol/L.

The mortality of CAP patients in ED who had a qSOFA ≥ 2 or lactate ≥ 2 mmol/L was 37.7%, which was significant higher than those patients who had qSOFA < 2 or lactate < 2 mmol/L (5.2%) (Table 7). For the secondary outcomes, significant difference was also found between the two groups in terms of ICU admission (27.3% vs. 0.9%), mechanical ventilation (36.4% vs. 2.6%), and vasopressor use (46.4% vs. 5.2%). Moreover, Kaplan–Meier survival curve analyses revealed that the CAP patients in ED with admission qSOFA < 2 or lactate ≤ 2 mmol/L showed significantly prolonged survival than those patients with qSOFA ≥ 2 or lactate > 2 mmol/L (Log-rank *χ*^2^ = 59.825, *P* < 0.001) (Figure 6).

## 4. Discussion

CAP is one of the most common infections seen in ED with a wide spectrum of severity and pathogens. Delayed treatment may result in serious consequences and even death. Therefore, timely diagnosis, assessment of severity, and treatment can improve prognosis. Severity evaluation is of vital significance in the initial management of CAP. Various scoring systems of CAP exist. They differ from each other, and they are used as tools to aid clinical diagnosis and treatment; however, doctors should take clinical experience into consideration [2].

As far as the components are concerned, the CURB65 score is very similar to qSOFA score. It comprises of five variables and divided patients into three broad risk groups as follows: Scores 0-1: low risk for 30-day mortality; Score 2: intermediate risk of 30-day mortality; Scores 3–5: high risk of 30-day mortality [3]. It was primarily designed to predict mortality and identify low-risk patients potentially suitable for ambulatory management and has been widely utilized in patients with CAP [10]. The CURB65 score has been extensively validated and performed similarly to the PSI score in predicting 30-day mortality of CAP patients [11], although previous study revealed that CURB65 may be more suitable for identifying high-risk patients, while PSI had advantage in the identification of low-risk patients [5]. The simplicity of calculation of CURB65 demonstrated superiority over other complex severity scores utilized in crowded emergency rooms. Furthermore, the CRB65 score, which does not require a blood urea level, is more suitable for use in gross-roots hospitals. Previous research studies demonstrated that CURB65/CRB65 does not incorporate an assessment of oxygenation and thus underestimated the risk of death and severity of influenza pneumonia [2, 12, 13].

Comparatively, the PSI score consisted of 20 different parameters with different weights including demographics, comorbidities, and clinical and laboratory findings [4]. The PSI score is heavily weighted by age and comorbidities, which shows that it underestimates severity of young CAP patients and it is not advised to guide intensive care unit admission [13, 14]. Moreover, the underlying health conditions may strongly influence mortality based on pneumonia severity in aged populations [15]. A study by Zhang et al. demonstrated that PSI performed better than CURB65 for mortality prediction, while its discriminative power decreased with advancing age [16]. Regrettably, the complexity of PSI limited its clinical application in ED.

The stratified and prognostic performance of SOFA and qSOFA in CAP has not yet been evaluated in detail, and only few studies have investigated its application in CAP in ED. Our previous research proved that SOFA is superior to CURB65, PSI, qSOFA, and procalcitonin in predicting 28-day mortality, with an AUROC of 0.913 [17], while Kim reported AUROC of SOFA and qSOFA to be 0.83 and 0.81, respectively [18]. Comparatively, qSOFA only requires a few items and vital signs. Previous research findings proved that it presented better clinical usefulness as prompt tools for ED or nonrespiratory specialists [19, 20].

Serum lactate is a well-known prognostic marker for patients with sepsis, and initial ED lactate is reported to be a useful marker to risk-stratify critically ill patients presenting to ED [7]. Sepsis 3.0 guidelines recommend lactate > 2.0 mmol/L with the requirement of vasopressor use to maintain a mean arterial blood pressure of 65 mmHg as the new definition of septic shock [6]. Obtaining blood for measuring lactate is recommended by surviving sepsis campaign bundle update, and if lactate > 2 mmol/L, it should be remeasured within 2–4 hours to guide resuscitation [21]. Nonetheless, the prognostic role of lactate on CAP patients has not been well studied. Gwak et al. enrolled 397 CAP patients and proved that the initial lactate level is independently associated with mortality in hospitalized patients with CAP [22]. Chen and Li enrolled 1641 patients and investigated the predictive performance of lactate, CURB65, and the combination of lactate and CURB65 for predicting mortality and ICU admission in pneumonia patients in ED, while results indicated that lactate is superior to CURB65 in predicting mortality, hospitalization, and intensive care unit (ICU) admission and lactate-CURB65 combination improves the predictive value of single CURB65 [10]. A similar study by Frenzen and colleagues concluded that admission lactate levels significantly improved the prognostic value (need for mechanical ventilation, vasopressors, ICU admission, or hospital mortality) of CRB/CURB65 scores in CAP patients with an optimal cutoff value of 1.8 mmol/L [23]. Song et al. enrolled 443 patients with CAP in ED, and results showed that the AUROC of qSOFA and SOFA for prediction of mortality was 0.720 and 0.845, while the combination of qSOFA and lactate was not significantly different from SOFA [24].

To the best of our knowledge, not many studies have explored the prognostic prediction performance of qSOFA score and admission lactate in septic CAP patients in ED before. Our results revealed that qSOFA has the highest sensitivity and negative predictive value in predicting both primary (28-day mortality) and secondary outcomes (ICU admission, mechanical ventilation, and vasopressor use). qSOFA outperformed other single predictors (CURB65, CRB65, PSI, SOFA, and lactate) in predicting 28-day mortality, although the AUROC of qSOFA was not significantly different from SOFA. Similar to previous studies, the combination of qSOFA + lactate was proved comparable to full version of SOFA in predicting primary and secondary outcomes in our research [25, 26]. Moreover, qSOFA + lactate was not statistically significant from SOFA + lactate (*Z* = 1.187, *P*=0.235). Regrettably, unlike the results of previous study [24], our research indicated that qSOFA + lactate and SOFA + lactate did not improve the prognostic prediction performance of single qSOFA (*Z* = 1.886, *P*=0.059) or single SOFA (*Z* = 1.066, *P*=0.286), respectively. The relatively small sample size may explain the result heterogeneity between our research and previous studies. Nonetheless, in view of the simplicity and convenience of qSOFA, it can be a better choice as tools for prognostic evaluation of septic CAP patients in ED. Furthermore, in predicting both primary and secondary outcomes (28-day mortality, ICU admission, mechanical ventilation, and vasopressor use), the optimal cutoff value for qSOFA was 2 and optimal cutoff value for admission lactate was 2 mmol/L. Our study revealed that septic CAP patients in ED with qSOFA < 2 or lactate ≤ 2 mmol/L demonstrated significantly prolonged survival than those patients with qSOFA ≥ 2 or admission lactate > 2 mmol/L, which could help physicians in ED enhance their awareness when treating these kind of septic CAP patients. A qSOFA of 2 or more would be very helpful and useful in discriminating high-risk patients with a high mortality rate.

Our research is among the very few studies that have explored the prognostic performance of CURB65, CRB65, PSI, SOFA, qSOFA, and admission lactate at the same time. Our results highlighted the superiority of qSOFA and SOFA in predicting 28-day mortality, ICU admission, mechanical ventilation, and vasopressor use. However, there are some limitations in our study. First, the relatively small sample size and the retrospective and single-center design may result in selection bias and do not allow for analysis of all clinical data. Our results should be verified by more multicenter, prospective studies with larger sample size. Second, our research only enrolled septic patients with CAP in ED. These patients are of older age and with more complications, which could have a higher mortality rate and may influence the final results.

## 5. Conclusions

In conclusion, we analyzed the prognostic prediction value of CURB65, CRB65, PSI, SOFA, qSOFA, and admission lactate at the same time in patients with ED in our solitary center. We found that qSOFA and SOFA were superior to CURB65, CRB65, and PSI in predicting 28-day mortality, ICU admission, mechanical ventilation, and vasopressor use for septic CAP patients in ED. The prediction performance of the combination of qSOFA and admission lactate was comparable to full version of SOFA. qSOFA with admission lactate could be a convenient, valuable, and practical tool for prognostic prediction. Further multicenter studies with larger sample size are needed to validate our results.

## Figures and Tables

**Table 1:** Baseline characteristics of enrolled patients with CAP in ED.

	All cohort	Death	Survival	*P*	ICU admission	Non-ICU admission	*P*
*N* (%)	336	89 (26.5)	247 (73.5)		61 (18.2)	275 (81.8)	
Age (yrs)	76 (61, 84)	78 (67, 85)	75 (61, 83)	0.136	81 (70, 88)	74 (61, 83)	0.003
Male, *n* (%)	213 (63.4)	56 (62.9)	157 (63.6)	0.914	40 (65.6)	173 (62.9)	0.696
Comorbidities, *n* (%)							
COPD	40 (11.9)	12 (13.5)	28 (11.3)	0.592	16 (26.2)	24 (8.7)	<0.001
CDVD	48 (14.3)	18 (20.2)	30 (12.1)	0.062	14 (23.0)	34 (12.4)	0.033
CBVD	88 (26.2)	28 (31.5)	60 (24.3)	0.187	18 (29.5)	70 (25.5)	0.515
Diabetes	77 (22.9)	31 (34.8)	46 (18.6)	0.002	24 (39.3)	53 (19.3)	0.001
CRD	30 (8.9)	9 (10.1)	21 (8.5)	0.648	8 (13.1)	22 (8.0)	0.205
HBD	24 (7.1)	5 (5.6)	19 (7.7)	0.515	4 (6.6)	20 (7.3)	0.844
Healthy	28 (8.3)	6 (6.7)	22 (8.9)	0.526	8 (13.1)	20 (7.3)	0.135
Laboratory results							
WBC (×10^9^/L)	10.0 (6.7, 14.1)	11.0 (6.7, 15.9)	9.8 (6.8, 13.9)	0.406	11.7 (7.5, 15.9)	9.7 (6.6, 13.9)	0.046
HGB (g/L)	126 (115, 137)	123 (111, 134)	127 (117, 138)	0.125	123 ± 22	127 (117, 138)	0.367
HCT (%)	36.9 (31.8, 40.8)	34.7 ± 9.1	37.3 (32.9, 40.9)	0.037	35.5 ± 9.3	37.3 (32.5)	0.368
PLT (×10^9^/L)	184 (131, 251)	163 (123, 249)	191 (136, 251)	0.311	155 (123, 230)	191 (137, 254)	0.116
ALB (g/L)	36.2 (32.1, 39.1)	34.0 ± 5.9	36.7 (33, 39.3)	0.001	34.3 ± 6.0	36.5 (32.5, 39.1)	0.044
CREA (*μ*mol/L)	80.5 (60.8, 114.3)	92 (63.6, 140)	75.9 (60.4, 105.8)	0.011	95.5 (74.6, 150)	75.9 (60.2, 105)	<0.001
BUN (mmol/L)	7.4 (5.3, 11.0)	8.7 (6.0, 13.4)	6.8 (5.1, 10.4)	0.010	10.4 (6.7, 16.1)	6.8 (5.1, 10.4)	<0.001
AST (U/L)	30 (20, 56)	33 (23, 68)	30 (20, 53)	0.174	36 (23, 64)	30 (20, 54)	0.240
ALT (U/L)	20 (14, 37)	17 (12, 35)	21 (14, 37)	0.225	16 (11, 32)	21 (14, 38)	0.167
TBIL (*μ*mol/L)	14.8 (9.6, 23.0)	15.3 (10.3, 23.5)	14.3 (9.5, 22.7)	0.318	15.1 (9.6, 23.6)	14.7 (9.8, 22.9)	0.778
DBIL (*μ*mol/L)	6.4 (4.0, 10.6)	6.8 (4.4, 11.3)	6.1 (3.8, 10.2)	0.089	6.1 (4.3, 11.5)	6.4 (4.0, 10.4)	0.503
K^+^ (mmol/L)	3.8 (3.5, 4.2)	3.9 (3.5, 4.4)	3.8 (3.5, 4.2)	0.252	4.0 (3.6, 4.4)	3.8 (3.5, 4.2)	0.050
PaO_2_/FiO_2_	303 (262, 352)	285 (246, 328)	313 (273, 363)	<0.001	286 (241, 328)	310 (268, 361)	0.007
Vital signs							
SBP (mmHg)	132 (119, 139)	122 (97, 135)	134 (125, 143)	<0.001	122 (96, 135)	132 (122, 142)	<0.001
DBP (mmHg)	68 (65, 76)	63 (55, 70)	70 (66, 78)	<0.001	65 ± 11	69 (65, 76)	<0.001
HR (times/min)	86 (78, 94)	87 (79, 106)	86 (78, 94)	0.009	86 (79, 106)	86 (78, 94)	0.024
Severity scores							
CURB65	2 (2, 3)	3 (3, 4)	2 (1, 3)	<0.001	3 (3, 4)	2 (2, 3)	<0.001
CRB65	2 (1, 3)	3 (2, 3)	2 (1, 2)	<0.001	3 (2, 3)	2 (1, 2)	<0.001
PSI	128 ± 40	157 ± 35	120 ± 38	<0.001	174 (150, 191)	121 ± 36	<0.001
SOFA	3 (2, 5)	6 (4, 8)	3 (2, 4)	<0.001	7 (6, 9)	3 (2, 4)	<0.001
qSOFA	2 (1, 2)	3 (2, 3)	1 (1, 2)	<0.001	3 (2, 3)	1 (1, 2)	<0.001
Lactate (mmol/L)	1.4 (1.1, 2.1)	1.8 (1.3, 2.9)	1.3 (1.1, 1.8)	<0.001	2.5 (1.6, 4.2)	1.3 (1.1, 1.8)	<0.001

Data are presented as *n*, *n* (%), or median (*Q*_L_, *Q*_U_). CAP: community-acquired pneumonia; ED: emergency department; SCAP: severe community-acquired pneumonia; NSCAP: nonsevere community-acquired pneumonia; COPD: chronic obstructive pulmonary disease; CDVD: cardiovascular disease; CBVD: cerebrovascular disease; CRD: chronic renal disease; HBD: hepatobiliary disease; WBC: white blood cell; HGB: hemoglobin; HCT: hematocrit; PLT: platelet; ALB: albumin; CREA: creatinine; BUN: blood urea nitrogen; AST: aspartate aminotransferase; ALT: alanine aminotransferase; TBIL: total bilirubin; DBIL: direct bilirubin; SBP: systolic blood pressure; DBP: diastolic blood pressure; HR: heart rate; CURB65: confusion, urea > 7 mmol/L, respiratory rate ≥ 30/min, blood pressure < 90 mmHg systolic and/or ≤60 mmHg diastolic, and age ≥ 65 years; CRB65: confusion, respiratory rate ≥ 30/min, blood pressure < 90 mmHg systolic and/or ≤60 mmHg diastolic, and age ≥ 65 years; PSI: pneumonia severity index; SOFA: sequential organ failure assessment; qSOFA: quick sequential organ failure assessment.

**Table 2:** Baseline characteristics of enrolled patients with CAP in ED.

	All cohorts	MV	NMV	*P*	Use of vasopressors	Nonuse of vasopressors	*P*
*N* (%)	336	83 (24.7)	253 (75.3)		108 (32.1)	228 (67.9)	
Age (yrs)	76 (61, 84)	78 (67, 84)	76 (61, 84)	0.584	78 (66, 87)	75 (61, 83)	0.065
Male, *n* (%)	213 (63.4)	54 (60.7)	159 (64.4)	0.535	71 (65.7)	142 (62.3)	0.539
Comorbidities, *n* (%)							
COPD	40 (11.9)	14 (15.7)	26 (10.5)	0.194	15 (13.9)	25 (11.0)	0.440
CDVD	48 (14.3)	14 (15.7)	34 (13.8)	0.650	18 (16.7)	30 (13.2)	0.391
CBVD	88 (26.2)	20 (22.5)	68 (27.5)	0.352	30 (27.8)	58 (25.4)	0.649
Diabetes	77 (22.9)	15 (16.9)	62 (25.1)	0.112	35 (32.4)	42 (18.4)	0.004
CRD	30 (8.9)	9 (10.1)	21 (8.5)	0.648	12 (11.1)	18 (7.9)	0.334
HBD	24 (7.1)	6 (6.7)	18 (7.3)	0.864	4 (3.7)	20 (8.8)	0.092
Healthy	28 (8.3)	7 (7.9)	21 (8.5)	0.852	5 (4.6)	23 (10.1)	0.091
Laboratory results							
WBC (×10^9^/L)	10.0 (6.7, 14.1)	10.4 (6.7, 15.8)	9.7 (6.8, 13.9)	0.244	10.8 (6.9, 16.0)	9.8 (6.6, 13.9)	0.180
HGB (g/L)	126 (115, 137)	126 (115, 137)	126 (116, 137)	0.781	126 (113, 136)	127 (117, 138)	0.314
HCT (%)	36.9 (31.8, 40.8)	35.4 ± 9.2	37.2 (32.7, 40.7)	0.356	34.9 ± 9.1	37.3 (32.9, 40.9)	0.092
PLT (×10^9^/L)	184 (131, 251)	155 (117, 241)	192 (141, 251)	0.109	162 (123, 245)	193 (141, 252)	0.082
ALB (g/L)	36.2 (32.1, 39.1)	34 ± 6	36.7 (32.8, 39.3)	0.002	34.2 ± 5.7	36.8 (33.0, 39.4)	<0.001
CREA (*μ*mol/L)	80.5 (60.8, 114.3)	92.5 (71.2, 149.2)	75.5 (59.9, 104)	<0.001	92 (69, 145.4)	75.4 (60, 101.4)	<0.001
BUN (mmol/L)	7.4 (5.3, 11.0)	9.5 (6.5, 15.0)	6.8 (5.0, 10.3)	<0.001	9.0 (6.0, 13.6)	6.8 (5.1, 10.0)	<0.001
AST (U/L)	30 (20, 56)	36 (23, 81)	29 (20, 53)	0.097	33 (23, 75)	29 (20, 53)	0.122
ALT (U/L)	20 (14, 37)	17 (12, 45)	21 (14, 36)	0.694	18 (12, 40)	21 (14, 37)	0.220
TBIL (*μ*mol/L)	14.8 (9.6, 23.0)	16.1 (10.6, 25)	14 (9.5, 22.3)	0.164	16.0 (10.2, 25.9)	13.9 (9.5, 21.9)	0.089
DBIL (*μ*mol/L)	6.4 (4.0, 10.6)	7 (4.4, 12.3)	6.1 (3.9, 9.7)	0.037	7.0 (4.4, 12.0)	6.0 (3.8, 9.6)	0.020
K^+^ (mmol/L)	3.8 (3.5, 4.2)	4 (3.6, 4.5)	3.8 (3.5, 4.1)	0.006	4.0 (3.5, 4.3)	3.8 (3.5, 4.2)	0.220
PaO_2_/FiO_2_	303 (262, 352)	285 (244, 326)	313 (272, 363)	<0.001	287 (246, 333)	315 (274, 363)	0.002
Vital signs							
SBP (mmHg)	132 (119, 139)	122 (100, 135)	134 (125, 143)	<0.001	113 (97, 133)	135 (126, 145)	<0.001
DBP (mmHg)	68 (65, 76)	65 (55, 71)	70 (66, 78)	<0.001	62 (55, 69)	72 (68, 78)	<0.001
HR (times/min)	86 (78, 94)	86 (79, 104)	86 (78, 94)	0.033	89 (79, 106)	86 (78, 92)	<0.001
Severity scores							
CURB65	2 (2, 3)	3 (3, 4)	2 (1, 3)	<0.001	3 (2.5, 4)	2 (1, 3)	<0.001
CRB65	2 (1, 3)	3 (2, 3)	2 (1, 2)	<0.001	3 (2, 3)	2 (1, 2)	<0.001
PSI	128 ± 40	161 ± 37	120 ± 36	<0.001	156 ± 38	118 ± 36	<0.001
SOFA	3 (2, 5)	6 (5, 9)	3 (2, 4)	<0.001	6 (4, 8)	3 (2, 4)	<0.001
qSOFA	2 (1, 2)	3 (2, 3)	1 (1, 2)	<0.001	2 (2, 3)	1 (1, 2)	<0.001
Lactate (mmol/L)	1.4 (1.1, 2.1)	1.9 (1.3, 4.2)	1.4 (1.1, 1.8)	<0.001	2.3 (1.4, 4.1)	1.3 (1.0, 1.6)	<0.001

Data are presented as *n*, *n* (%), or median (*Q*_L_, *Q*_U_). CAP: community-acquired pneumonia; MV: mechanical ventilation; NMV: nonmechanical ventilation; ED: emergency department; SCAP: severe community-acquired pneumonia; NSCAP: nonsevere community-acquired pneumonia; COPD: chronic obstructive pulmonary disease; CDVD: cardiovascular disease; CBVD: cerebrovascular disease; CRD: chronic renal disease; HBD: hepatobiliary disease; WBC: white blood cell; HGB: hemoglobin; HCT: hematocrit; PLT: platelet; ALB: albumin; CREA: creatinine; BUN: blood urea nitrogen; AST: aspartate aminotransferase; ALT: alanine aminotransferase; TBIL: total bilirubin; DBIL: direct bilirubin; SBP: systolic blood pressure; DBP: diastolic blood pressure; HR: heart rate; CURB65: confusion, urea > 7 mmol/L, respiratory rate ≥ 30/min, blood pressure < 90 mmHg systolic and/or ≤60 mmHg diastolic, and age ≥ 65 years; CRB65: confusion, respiratory rate ≥ 30/min, blood pressure < 90 mmHg systolic and/or ≤60 mmHg diastolic, and age ≥ 65 years; PSI: pneumonia severity index; SOFA: sequential organ failure assessment; qSOFA: quick sequential organ failure assessment.

**Table 3:** ROC curve comparisons between CAP severity scores and admission lactate in predicting 28-day mortality.

	AUC (95% CI)	*P* value	Cutoff value	Sensi (%)	Speci (%)	PPV (%)	NPV (%)
CURB65	0.744 (0.685–0.802)	<0.001	3.0	75.3	61.9	41.6	87.4
CRB65	0.737 (0.675–0.799)	<0.001	3.0	56.2	83.0	54.4	84.0
PSI	0.768 (0.711–0.824)	<0.001	131	77.5	64.0	43.7	88.8
SOFA	0.795 (0.740–0.850)	<0.001	4.0	84.3	64.4	46.0	91.9
qSOFA	0.807 (0.754–0.859)	<0.001	2.0	91.0	53.8	41.5	94.3
Lactate	0.679 (0.612–0.745)	<0.001	2.0	48.3	78.1	44.3	80.7
CURB65 + lactate	0.772 (0.718–0.827)	<0.001	0.18	83.1	59.5	42.5	90.7
CRB65 + lactate	0.776 (0.719–0.833)	<0.001	0.25	68.5	77.7	52.5	87.3
PSI + lactate	0.774 (0.719–0.828)	<0.001	0.21	80.9	61.9	43.3	90.0
SOFA + lactate	0.803 (0.749–0.857)	<0.001	0.23	74.2	76.5	53.2	89.2
qSOFA + lactate	0.833 (0.786–0.881)	<0.001	0.29	64.0	87.9	65.6	87.1

ROC: area under the curve; CAP: community-acquired pneumonia; Sensi: sensitivity; Speci: specificity; PPV: positive predictive value; NPV: negative predictive value; CURB65: confusion, urea > 7 mmol/L, respiratory rate ≥ 30/min, blood pressure < 90 mmHg systolic and/or ≤60 mmHg diastolic, and age ≥ 65 years; CRB65: confusion, respiratory rate ≥30/min, blood pressure < 90 mmHg systolic and/or ≤60 mmHg diastolic, and age ≥ 65 years; PSI: pneumonia severity index; SOFA: sequential organ failure assessment; qSOFA: quick sequential organ failure assessment.

**Table 4:** ROC curve comparisons among severity scores and admission lactate in predicting ICU admission.

	AUC (95% CI)	*P* value	Cutoff value	Sensi (%)	Speci (%)	PPV (%)	NPV (%)
CURB65	0.774 (0.709–0.840)	<0.001	3.0	83.6	60.0	31.7	94.3
CRB65	0.738 (0.667–0.810)	<0.001	3.0	59.0	79.6	39.1	89.7
PSI	0.837 (0.777–0.896)	<0.001	155	73.8	83.6	49.9	93.5
SOFA	0.895 (0.846–0.943)	<0.001	6.0	77.0	90.2	63.5	94.6
qSOFA	0.822 (0.769–0.874)	<0.001	2.0	96.7	50.5	30.2	98.6
Lactate	0.742 (0.669–0.816)	<0.001	2.0	55.7	77.1	35.0	88.7
CURB65 + lactate	0.818 (0.759–0.877)	<0.001	0.21	67.2	82.2	45.6	91.9
CRB65 + lactate	0.806 (0.741–0.870)	<0.001	0.16	77.0	74.9	40.5	93.6
PSI + lactate	0.840 (0.782–0.898)	<0.001	0.25	72.1	86.2	53.7	93.3
SOFA + lactate	0.902 (0.857–0.947)	<0.001	0.18	82.0	87.6	59.5	95.6
qSOFA + lactate	0.868 (0.823–0.912)	<0.001	0.14	83.6	78.2	46.0	95.6

ROC: area under the curve; CAP: community-acquired pneumonia; SCAP: severe community-acquired pneumonia; Sensi: sensitivity; Speci: specificity; PPV: positive predictive value; NPV: negative predictive value; CURB65: confusion, urea > 7 mmol/L, respiratory rate ≥ 30/min, blood pressure < 90 mmHg systolic and/or ≤60 mmHg diastolic, and age ≥ 65 years; CRB65: confusion, respiratory rate ≥ 30/min, blood pressure < 90 mmHg systolic and/or ≤60 mmHg diastolic, and age ≥ 65 years; PSI: pneumonia severity index; SOFA: sequential organ failure assessment; qSOFA: quick sequential organ failure assessment.

**Table 5:** ROC curve comparisons between CAP severity scores and admission lactate in predicting mechanical ventilation.

	AUC (95% CI)	*P* value	Cutoff value	Sensi (%)	Speci (%)	PPV (%)	NPV (%)
CURB65	0.771 (0.713–0.829)	<0.001	3.0	78.3	62.1	40.4	89.7
CRB65	0.758 (0.697–0.820)	<0.001	3.0	59.0	83.0	53.2	86.1
PSI	0.780 (0.722–0.837)	<0.001	150	63.9	80.6	51.9	87.2
SOFA	0.845 (0.794–0.895)	<0.001	5.0	75.9	78.7	53.9	90.9
qSOFA	0.838 (0.789–0.887)	<0.001	2.0	94.0	53.8	40.0	96.5
Lactate	0.698 (0.628–0.768)	<0.001	2.0	49.4	77.9	42.3	82.4
CURB65 + lactate	0.804 (0.752–0.857)	<0.001	0.26	63.9	80.6	51.9	87.2
CRB65 + lactate	0.809 (0.755–0.864)	<0.001	0.21	75.9	76.7	51.7	90.7
PSI + lactate	0.796 (0.741–0.850)	<0.001	0.23	71.1	75.1	48.4	88.8
SOFA + lactate	0.851 (0.801–0.902)	<0.001	0.21	79.5	77.9	54.1	92.1
qSOFA + lactate	0.868 (0.826–0.911)	<0.001	0.26	71.1	88.9	67.8	90.4

ROC: area under the curve; CAP: community-acquired pneumonia; Sensi: sensitivity; Speci: specificity; PPV: positive predictive value; NPV: negative predictive value; CURB65: confusion, urea > 7 mmol/L, respiratory rate  ≥ 30/min, blood pressure < 90 mmHg systolic and/or ≤60 mmHg diastolic, and age ≥ 65 years; CRB65: confusion, respiratory rate≥30/min, blood pressure < 90 mmHg systolic and/or ≤60 mmHg diastolic, and age ≥ 65 years; PSI: pneumonia severity index; SOFA: sequential organ failure assessment; qSOFA: quick sequential organ failure assessment.

**Table 6:** ROC curve comparisons between CAP severity scores and admission lactate in predicting vasopressor use.

	AUC (95% CI)	*P* value	Cutoff value	Sensi (%)	Speci (%)	PPV (%)	NPV (%)
CURB65	0.759 (0.705–0.814)	<0.001	3.0	75.0	64.9	50.3	84.6
CRB65	0.746 (0.688–0.803)	<0.001	3.0	53.7	85.1	63.1	79.5
PSI	0.767 (0.712–0.823)	<0.001	150.0	60.2	83.8	63.8	81.6
SOFA	0.821 (0.772–0.871)	<0.001	4.0	84.3	68.4	55.8	90.2
qSOFA	0.820 (0.772–0.868)	<0.001	2.0	90.7	57.5	50.3	92.9
Lactate	0.758 (0.700–0.817)	<0.001	2.0	57.4	84.6	68.7	81.3
CURB65 + lactate	0.824 (0.778–0.871)	<0.001	0.24	84.3	67.1	54.8	90.0
CRB65 + lactate	0.830 (0.782–0.877)	<0.001	0.31	75.0	80.3	64.3	87.1
PSI + lactate	0.820 (0.773–0.867)	<0.001	0.28	76.9	76.3	60.6	87.5
SOFA + lactate	0.848 (0.801–0.894)	<0.001	0.29	75.0	82.5	67.0	87.4
qSOFA + lactate	0.875 (0.835–0.915)	<0.001	0.38	69.4	89.9	76.5	86.1

ROC: area under the curve; CAP: community-acquired pneumonia; Sensi: sensitivity; Speci: specificity; PPV: positive predictive value; NPV: negative predictive value; CURB65: confusion, urea > 7 mmol/L, respiratory rate ≥ 30/min, blood pressure < 90 mmHg systolic and/or ≤60 mmHg diastolic, and age ≥ 65 years; CRB65: confusion, respiratory rate ≥ 30/min, blood pressure < 90 mmHg systolic and/or ≤60 mmHg diastolic, and age ≥ 65 years; PSI: pneumonia severity index; SOFA: sequential organ failure assessment; qSOFA: quick sequential organ failure assessment.

**Table 7:** Comparisons of severity scores and different outcomes in patients with CAP using qSOFA and lactate.

	qSOFA ≥ 2 or lactate > 2		*P* value
	Yes (*n* = 216)	No (*n* = 120)	
Severity scores			
CURB65	3 (2, 4)	2 (1, 2)	<0.001
CRB65	2 (2, 3)	1 (1, 2)	<0.001
PSI	144 ± 37	104 ± 32	<0.001
SOFA	4 (3, 6)	2 (2, 3)	<0.001
qSOFA	2 (2, 3)	1 (1, 1)	<0.001
Lactate	1.8 (1.2, 2.8)	1.2 (1.0, 1.4)	<0.001

Primary outcome, *n* (%)			
28-day mortality	83 (38.4)	6 (5.0)	<0.001

Secondary outcome, *n* (%)			
ICU admission	60 (27.8)	1 (0.8)	<0.001
Mechanical ventilation	80 (37.0)	3 (2.5)	<0.001
Use of vasopressors	102 (47.2)	6 (5.0)	<0.001

Data are presented as *n*, *n* (%), or median (*Q*_L_, *Q*_U_). CAP: community-acquired pneumonia; CURB65: confusion, urea > 7 mmol/L, respiratory rate ≥ 30/min, blood pressure < 90 mmHg systolic and/or ≤60 mmHg diastolic, and age ≥ 65 years; CRB65: confusion, respiratory rate ≥ 30/min, blood pressure < 90 mmHg systolic and/or ≤60 mmHg diastolic, and age ≥ 65 years; PSI: pneumonia severity index; SOFA: sequential organ failure assessment; qSOFA: quick sequential organ failure assessment; ICU: intensive care unit.

## Data Availability

The data used to support the findings of this study are available from the corresponding author upon request

## Abbreviations

CAP: Community-acquired pneumonia
ED: Emergency department
PSI: Pneumonia severity index
SOFA: Sequential organ failure assessment
CURB65: Confusion, urea > 7 mmol/L, respiratory rate ≥ 30/min, blood pressure <90 mmHg systolic and or ≤60 mmHg diastolic, and age ≥ 65 years
CRB65: Confusion, respiratory rate ≥ 30/min, blood pressure <90 mmHg systolic and/or ≤60 mmHg diastolic, and age ≥ 65 years
HGB: Hemoglobin
HCT: Hemocrit
PLT: Platelet
ALB: Albumin
AST: Aspartate aminotransferase
ALT: Alanine aminotransferase
TBIL: Total bilirubin
DBIL: Direct bilirubin
CREA: Creatinine
BUN: Blood urea nitrogen
WBC: White blood cell
ROC: Receiver operating characteristics
AUC: Area under the curve
AUROC: Area under the receiver operating characteristics curve
PPV: Positive predictive value
NPV: Negative predictive value
COPD: Chronic obstructive pulmonary disease
CDVD: Cardiovascular disease
CBVD: Cerebral-vascular disease
CRD: Chronic renal disease
HBD: Hepatobiliary disease
ICU: Intensive care unit.
